# A New Species of *Aprostocetus* (Hymenoptera: Eulophidae), a Parasitoid from China of the Invasive Gall Wasp *Ophelimus bipolaris* (Hymenoptera: Eulophidae) on *Eucalyptus*

**DOI:** 10.3390/insects16080755

**Published:** 2025-07-23

**Authors:** Jing-Hui Su, Yuan-Hao Li, Jin Hu, Yan Qin, Jun Li, Zoya Yefremova, Xia-Lin Zheng

**Affiliations:** 1 Guangxi Key Laboratory of Agro-Environment and Agric-Products Safety, College of Agriculture, Guangxi University, Nanning 530004, China; sjh20022@126.com (J.-H.S.); l18137818978@163.com (Y.-H.L.); 18851822718@163.com (J.H.); lijunlijun1981@163.com (J.L.); 2 Guangxi Zhuang Autonomous Region State-Owned Qipo Forest Farm, Nanning 530031, China; gxqinyan@163.com; 3 The Steinhardt Museum of Natural History, Israel National Center for Biodiversity Studies and Department of Zoology, Tel Aviv University, Tel Aviv 6997801, Israel

**Keywords:** Chalcidoidea, Opheliminae, Tetrastichinae, natural enemy, COI gene, 28S gene, integrative taxonomy

## Abstract

A new species of *Aprostocetus* (Hymenoptera: Eulophidae), *Aprostocetus bipolaris* sp. nov., is recognized to be fortuitously present on a population of the invasive *Eucalyptus* (*E. grandis* × *E. urophylla*) gall wasp *Ophelimus bipolaris* Chen & Yao, in Guangxi, China. To classify this species, an integrated approach of morphological characteristics and molecular data was applied. The morphology of the new species is described and illustrated, and an identification key for female and male adults is also presented. Regarding phylogenetic analyses, the position of *A. bipolaris* sp. nov. within the *Aprostocetus* group of genera was reaffirmed based on *28S* and *COI* gene sequences. All these lines of evidence indicate that *A. bipolaris* sp. nov. is a new species.

## Simple Summary

*Aprostocetus bipolaris* sp. nov. (Hymenoptera: Eulophidae), a newly described parasitoid species, was discovered in Guangxi of China in association with the invasive gall-forming pest *Ophelimus bipolaris* on *Eucalyptus*. The new species is described and illustrated, and an updated key to the female and male adults is provided. Molecular analyses are also conducted using *28S* and *COI* gene sequences. The taxonomic decision of the new parasitoid species was based on morphological and molecular evidence. The diagnostic characters used for species identification and potential applications in biological control of this species are discussed.

## 1. Introduction

The genus *Ophelimus* (Eulophidae) is native to Australia and is among the most important invasive species in *Eucalyptus* plantations in the world [1,2]. Species of this genus induce galls on various species of *Eucalyptus* (Myrtaceae) [1]. A large number of galls induced by *Ophelimus* spp. can cause severe defoliation, weaken trees, or stunt the growth of eucalypts, eventually resulting in significant economic losses [3,4,5,6,7]. To date, over 50 species of this genus have been described [8]. Among these species, the first *Eucalyptus* gall wasp, *O. eucalypti* (Gahan), recorded outside Australia was in Wellington, New Zealand in 1922 [9]. Originally, it was described as *Rhicnopeltella eucalypti* Gahan and was transferred to the genus *Ophelimus* by Bouček [1]. Currently, *Ophelimus* species have invaded different areas worldwide, and *O. maskelli* (Ashmead) is the most widely distributed [10,11,12,13].

Recently, a new invasive *Eucalyptus* gall wasp, *O. bipolaris* Chen & Yao, was found in China, and its origin was considered to be Australia or Indonesia [14]. Similar to other *Eucalyptus* gall wasps, *O. bipolaris* induces galls on various species of *Eucalyptus*, with galls characteristically protruding from both leaf surfaces. Galls change from green to red, then to brown. Following adult emergence, galls exhibit characteristic circular exit holes. Populations exhibit a pronounced female-biased sex ratio, completing their life cycle within approximately two months under field conditions.

Biological control is considered to have long-term ecological and economic benefits in terms of controlling exotic pests [15]. At present, a few natural enemy species of the genus *Ophelimus* have been recorded, including *Closterocerus chamaeleon* (Girault) (Eulophidae), *Stethynium ophelimi* Huber, *S. breviovipositor* Huber (Mymaridae), *Aprostocetus causalis* La Salle & Wu, *Aprostocetus* sp., *Chrysonotomyia germanica* Erdös, *Chrysonotomyia* sp., *Eurytoma* sp. (Eurytomidae), and *Quadrastichus mendeli* Kim & La Salle [11,16,17]. Among them, *C. chamaeleon* is vastly reported as an efficacious bio-controller of *O. maskelli* around the world [4,18,19,20,21]. However, some *Ophelimus* species can escape the parasitoidism by *C. chamaeleon* through an asynchronous life cycle in this host–parasitoid system [5].

The genus *Aprostocetus* (Eulophidae) was first described by Westwood [22] and now comprises over 800 described species [8]. Many *Aprostocetus* species are primary parasitoids of hosts in plant galls [23,24,25], such as Aphalaridae [26], Cecidomyiidae [27,28], Blattidae [29], and Pseudococcidae [30]. Some species also parasitize members of the Eulophidae. For instance, *A. causalis* parasitizes *Leptocybe invasa* Fisher & La Salle on *Eucalyptus* spp. in China and Thailand, while *A. felix* La Salle, Yang & Lin targets *Quadrastichus erythrinae* Kim on *Erythrina* spp. (Fabaceae) in Taiwan of China [31]. The earliest record of the genus in China dates to 1921, when Perkins documented *A. muiri* during his study of sugarcane pest, parasites, and predators [32]. Up to now, many species from the *Aprostocetus* have been utilized in numerous countries for the biological control of pests [33,34,35,36,37]. A notable example is *A. fukutai* (Miwa & Sonan), which was accidentally introduced from East Asia to Italy as a biological control agent for managing the citrus long-horned beetle *Anoplophora chinensis* (Forster) [38].

**Figure 1:**
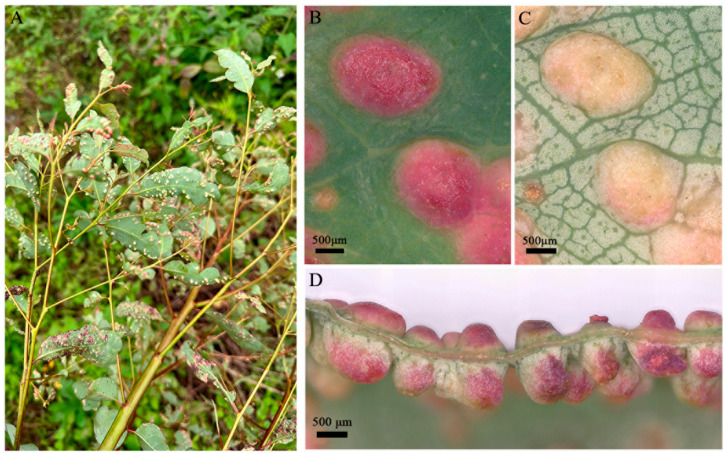
Galls induced by *Ophelimus bipolaris* on *Eucalyptus grandis* × *Eucalyptus urophylla*. (**A**) Galls induced on leaves; (**B**) mature galls on the leaf, upper view; (**C**) mature galls on the leaf, ventral view; (**D**) mature galls on the leaf, lateral view.

In August 2023, we found hybrid *Eucalyptus* (*E. grandis* Hill ex maiden × *E. urophylla* S. T. Blake) leaves that had been galled by *O. bipolaris* (Figure 1) in Hechi City, Guangxi Zhuang Autonomous Region, China. Some infested branches with mature galls induced by *O. bipolaris* were collected and brought back to our laboratory. An unknown parasitoid emerged from the galls and was preliminary identified as a *Aprostocetus* species. In addition, field data show that its parasitoidism rate is 18.52% (unpublished data). In the present study, to identify the unknown parasitoid, we conducted identification using morphology and molecular analysis.

## 2. Materials and Methods

### 2.1. Insect Sampling

Infested branches with mature galls (Figure 1) induced by *O. bipolaris* were collected in August 2023 in Hechi City (108°14′18′′ E, 25°21′1′′ N), Guangxi Zhuang Autonomous Region, China. Branches were placed in a plastic container (height × diameter = 15 cm × 13 cm) filled with water to retain their freshness, and they were then transferred to a sealed net cage (length × width × height = 40 cm × 40 cm × 80 cm). Branches were maintained at 26 ± 1 °C, 70–80% relative humidity with a photoperiod of 13:11 h (light: dark) for adult emergence. The net cage was checked daily, and emerged adults landing on the inner wall of the net cage were captured using 1.5 mL centrifuge tubes. All adults were maintained in centrifuge tubes, killed in 95% ethanol, and then used for morphological and molecular identification.

The examined specimens are deposited in the College of Agriculture, Guangxi University (GXU).

### 2.2. Morphology

Morphological terminology used in this paper follows Burks et al. [39]. The following acronyms are applied to morphology: F1–F4, first, second, third and fourth funicular segments of antenna; SMV, submarginal; MV, marginal; PMV, postmarginal; STV, stigmal veins; C1–C3, first, second, and third claval segments; POL, postocellar line; the minimum distance between the posterior ocelli; OOL, ocellocular line; Gt1–Gt3, first, second and third gastral tergum.

### 2.3. Measurements

Specimens preserved in 95% ethanol were carefully removed and blotted on filter paper to eliminate excess alcohol. For general morphological measurements, intact specimens were directly positioned under a microscope in their ethanol-preserved state. For specialized examination of ovipositors and genitalia, specimens were first blotted dry and then transferred to glass slides with a drop of distilled water. Following dissection, the isolated reproductive structures were covered with a cover slip and immediately imaged under a microscope. Absolute measurements in micrometers (μm) were used for the body, forewing, ovipositor, and genitalia length. For other dimensions, relative measurements were used.

### 2.4. Images

A digital microscope VHX-6000 (Keyence, Osaka, Japan) and scanning electron microscope FEI Quattro S (Thermo Fisher Scientific, Czech Republic) were used for identification and photography.

### 2.5. DNA Extraction, Amplification, and Sequencing

DNA was extracted from adult specimens using the TIANamp Genomic DNA Kit (DP304-02, TianGen, Beijing, China) following the manufacturer’s protocol.

For genetic characterization, the *28S* ribosomal RNA gene (*28S* rRNA) and cytochrome c oxidase subunit I gene (*COI*) were selected as targets. Subsequently, the *28S* gene was amplified using the primers D2-3551F (5′-CGTGTTGCTTGATAGTGCAGC-3′) and D2-4057R (5′-TCAAGACGGGTCCTG AAAGT-3′) [40]. The *COI* gene was amplified using primers LCO1490F (5′-GTCAACAAATCATAAAGATATTGG-3′) and HCO2198R (5′-TAAACTTCAGGGTGACCAAAAAATCA-3′) [41].

The PCR reactions were conducted in a T100™ Thermal Cycler (Bio-Rad Laboratories Pty. Ltd., Singapore) with a total reaction volume of 50 μL, containing 25 μL of premix Taq polymerase (RR902A, Takara, Dalian, China), 20 μL of ddH_2_O, 1.5 μL of each primer (10 pmol/μL), and 2 μL of extracted DNA. Thermocycling conditions were an initial denaturing step at 94 °C for 3 min, followed by 35 cycles of 30 s at 94 °C, 30 s at 53 °C for *28S*, 49 °C for *COI*, and 1 min at 72 °C. A final elongation at 72 °C for 5 min. The PCR products were sequenced in both directions at Sangon Biotech Co., Ltd. (Shanghai, China).

### 2.6. Sequence Alignments and Phylogenetic Analysis

The obtained sequences were assembled and analyzed using DNAMAN V6 software. After the analysis, all the sequences were submitted to GenBank (http://www.ncbi.nlm.nih.gov) to obtain accession numbers (Table 1). Subsequently, the NCBI Nucleotide Blast tool (https://blast.ncbi.nlm.nih.gov/Blast.cgi, accessed on 21 July 2025) was employed to search for similar sequences.

Afterwards, multiple alignments of nucleotide sequences were performed using ClustalW V2 [42]. MEGA V7 software was used to perform the phylogenetic analysis [43]. The maximum likelihood (ML) trees were reconstructed with 1000 bootstrap replications [44,45].

## 3. Results

### 3.1. Taxonomy

**Figure 2:**
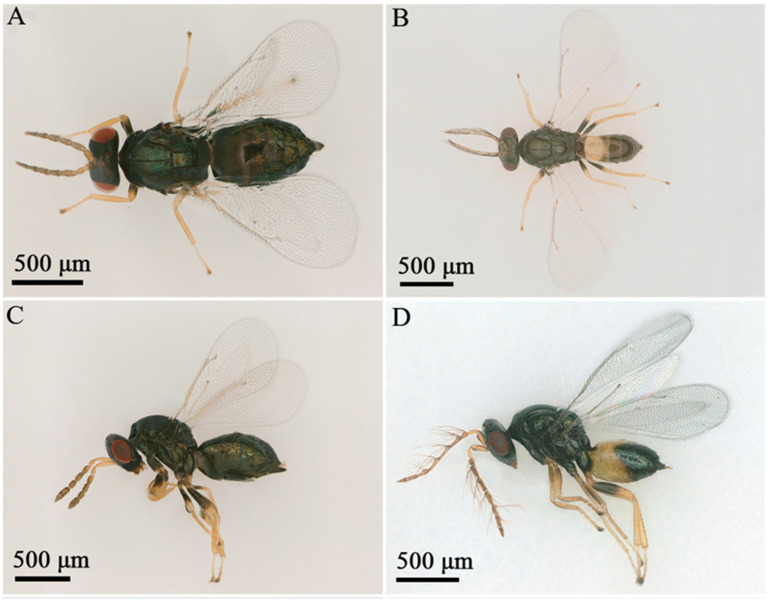
*Aprostocetus bipolaris* sp. nov. female (left column) and male (right column). (**A**,**B**) Dorsal view; (**C**,**D**) lateral view.

**Figure 3:**
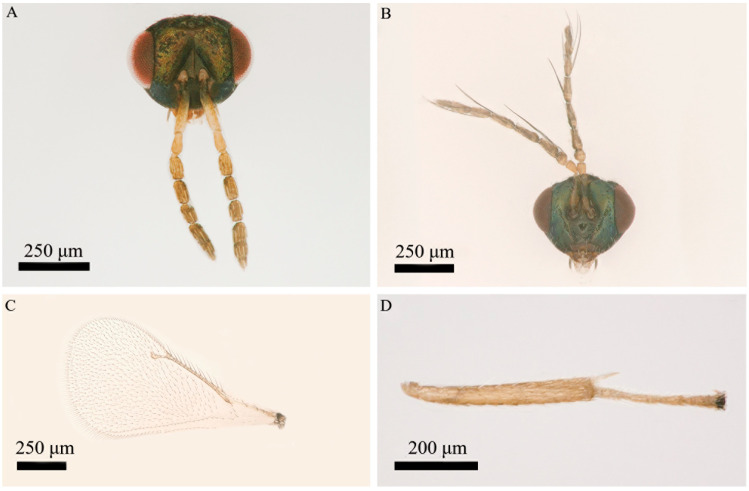
*Aprostocetus bipolaris* sp. nov. (**A**) Head, frontal (female); (**B**) head, frontal (male); (**C**) fore wing; (**D**) mid tibia with one spur.

**Figure 4:**
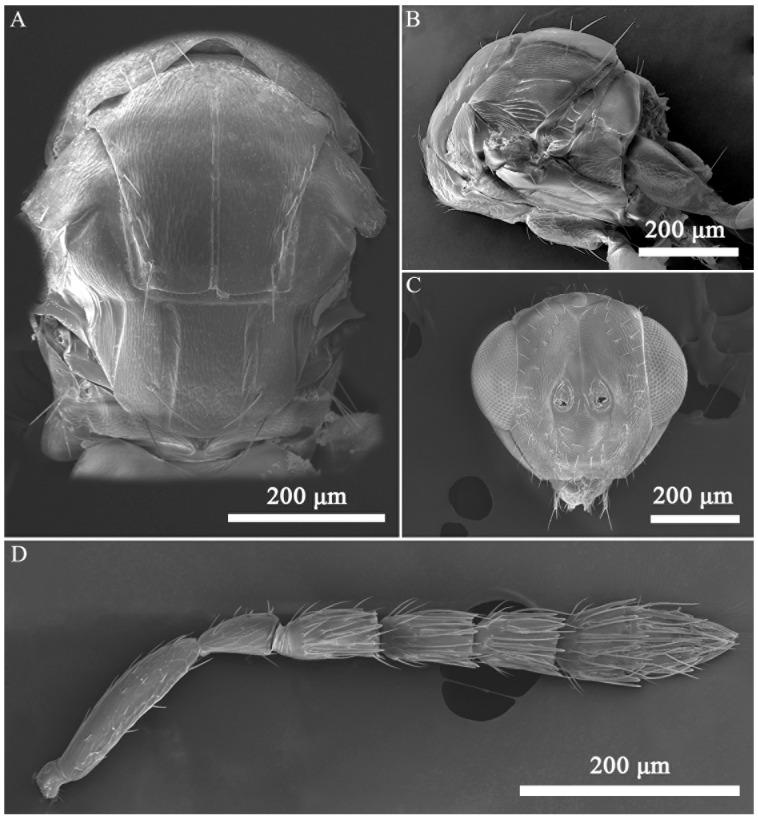
*Aprostocetus bipolaris* sp. nov., female. (**A**) Mesosoma, dorsal view; (**B**) mesosoma, lateral view; (**C**) head, frontal; (**D**) antenna.

**Figure 5:**
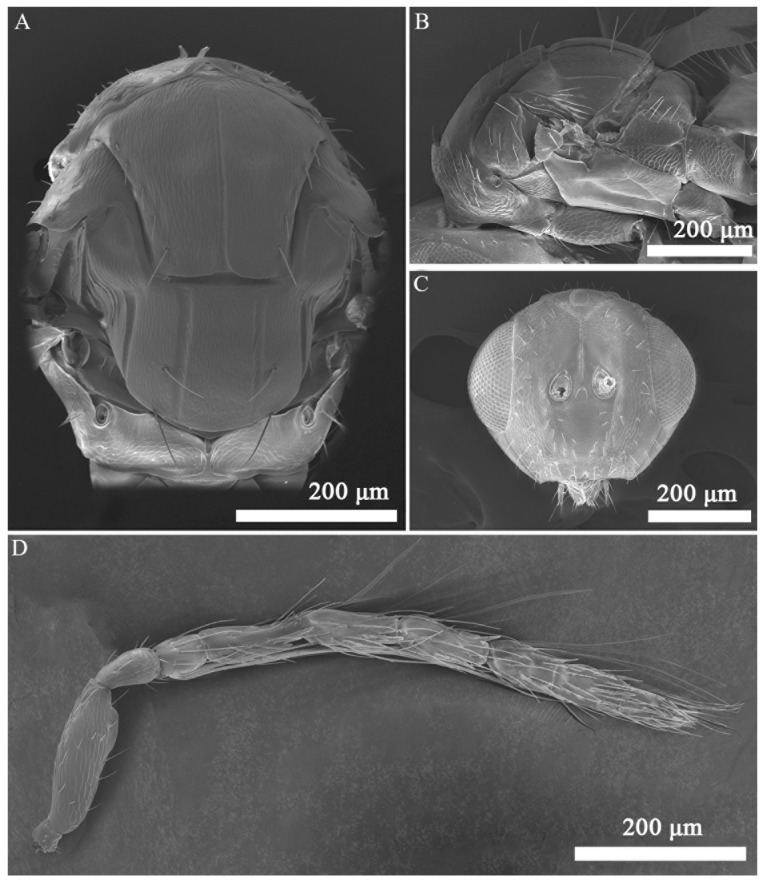
*Aprostocetus bipolaris* sp. nov., male. (**A**) Mesosoma, dorsal view; (**B**) mesosoma, lateral view; (**C**) head, frontal; (**D**) antenna.

**Figure 6:**
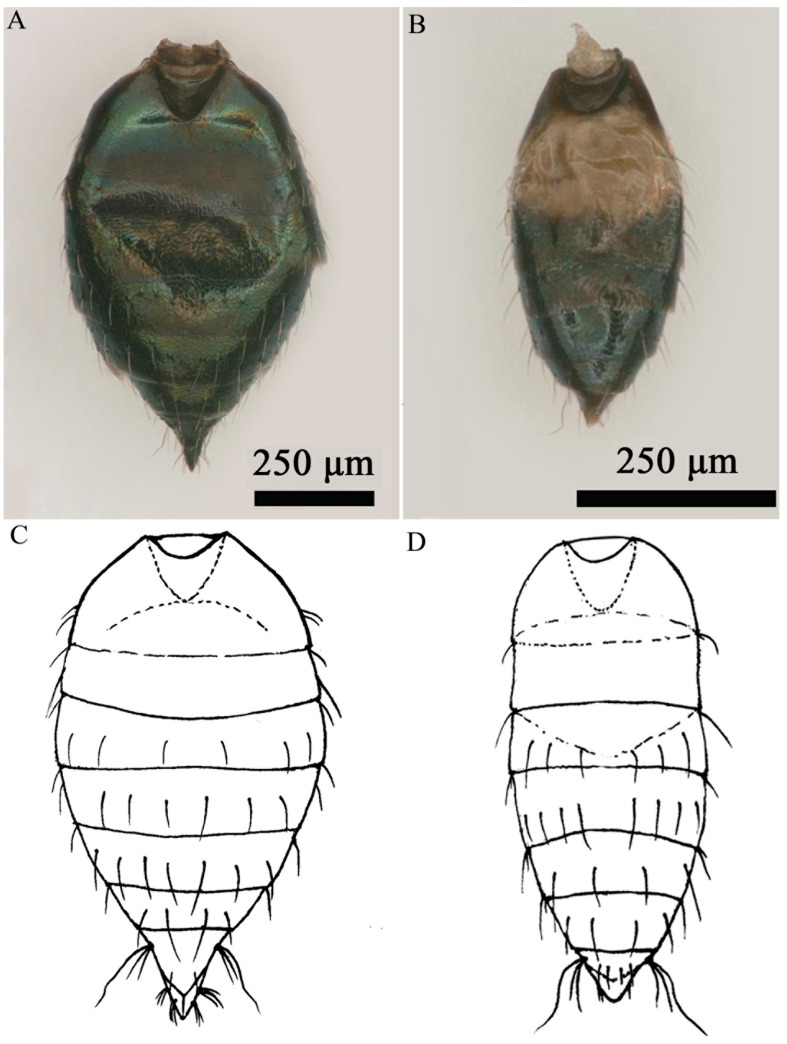
*Aprostocetus bipolaris* sp. nov. (**A**,**C**) Gaster, dorsal view (female); (**B**,**D**) gaster, dorsal view (male).

**Figure 7:**
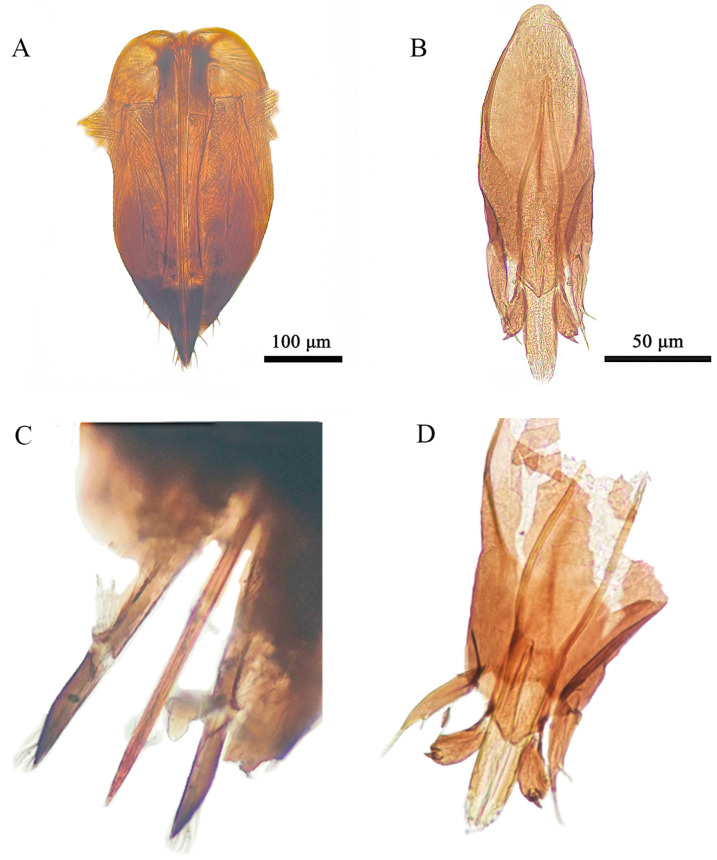
*Aprostocetus bipolaris* sp. nov. (**A**) Ovipositor; (**B**) genitalia; (**C**) apex of ovipositor: two ovipositors sheaths and ovipositor stilets; (**D**) genitalia ventral: aedeagus with aedeagal apodemae, phallobase, volsellar digiti with digital spines and parameres.

*Aprostocetus bipolaris* Zheng & Yefremova sp. nov. (Figure 2, Figure 3, Figure 4, Figure 5, Figure 6 and Figure 7).

LSID urn:lsid:zoobank.org:pub:030F1F20-E68E-489A-9D18-5901166768AD.

Diagnosis (Figure 3, Figure 4, Figure 5, Figure 6 and Figure 7). SMV with 3 setae. Mesoscutum with a median groove and five adnotaular setae in one row from both lateral sides. Propodeal callus with four setae in two rows. Propodeum 1.2× as long as dorsellum. Female. Antenna with scape 4.1× as long as broad, F1 = F2, F3 0.88× as long as F2. Clava 2.0× as long as F3. Gaster 1.6× as long as broad. Cercus with three setae, the longest 1.93× as long as the next seta. Male. Antennal ventral plaque 0.33 of length of scape. F1 0.5× as long as F2. F2 = F3 = F4. Clava 2.4× as long as F4. Whorls of F1 and F2 short, whorls of F4 reaching base of C2 and whorls of C1 reaching apex of C3. Gaster 2.0× as long as broad. Cercus with six setae, the longest seta kinked, and 2.35× as long as next seta. The genitalia, digiti with one developed spine.

Descriptions. Female (Figure 2A,C): Body length 1263.44–1821.54 μm.

Head (Figure 3A and Figure 4C) 1.2× as long as long as broad; POL 1.6× as long as OOL. Clypeus bidentate. Frons with median longitudinal line. Mandibles with two teeth. Eye without hair. Malar sulcus straight. Breadth of mouth opening 1.5× of the malar space length. Antenna (Figure 4D) with scape 4.1× as long as broad, pedicel 1.8× as long as broad, one anellus, F1 1.9× as long as broad, F2 1.9× as long as broad, F3 1.6× as long as broad, clava 3-segmented 2.0× as long as broad. F1 = F2, F3 0.88× as long as F2. Clava 2.1× as long as F3.

Mesosoma (Figure 4A). Mesoscutum 1.2× as broad as long, with a distinct median line and 5 adnotaular setae in one row from both lateral sides. Scutellum 1.3× as broad as long, with two pairs of setae. Anterior setae on the lower level of anterior half of scutellum and 0.77× as long as posterior setae (Figure 4B). Distance between sublateral and submedian lines 1.2× as long as distance between submedian and lateral lines. Dorsellum 0.83× as long as propodeum. Propodeum 6.0× as broad as long, spiracles rounded, with a postspiracle rim, placed near frontal margin of propodeum, superfacial reticulate; callus with four setae in two rows. Forewing (Figure 3C) 2.4× as long as broad. SMV:MV:STV = 25:40:10. MV 1.5× as longer as costal cell. PMV stub. Speculum closed and extended ¼ below MV. SMV with three setae. MV with nine setae. Stigma of STV with three short setae.

Metasoma. Petiole transverse, small, smooth. Gaster (Figure 6A) 1.6× as long as broad; last tergite 3.5× as broad as long, cercus with three setae, the longest cercal seta kinked and 1.93× as long as the next seta (Figure 6C). Ovipositor sheaths (Figure 6A) slightly extended (n = 15). Sheaths (Figure 7C) covered by several setae in apical part. The ratio of length of ovipositor and length of ovipositor sheath = 4.8:1 (n = 8) (Figure 7A).

Color (Figure 2 and Figure 3). Head, vertex, and occiput metallic green. Eye red. Ocelli yellow. Antenna flagellum brown, scape and pedicel yellow. Mesosoma dark brown, with scutellum and dorsellum exhibiting dark brown coloration with green tint. Mesosoma, scutellum, dorsellum dark brown with green tint. Legs yellow with dark brown coxae, dorsally brown femora and brown last tarsal segment. Gaster dark brown.

Male (Figure 2B,D): Body length 1145.33–1704.26 μm.

Similar to female, except: Face is covered with numerous setae in lower part (Figure 5C) more than female (Figure 4C). Breadth of mouth opening 2.0× the malar sulcus. Antenna (Figure 5D) with scape 3.5× as long as broad, ventral plaque 0.33 of length of scape and placed in upper part. Pedicel 1.3× as long as broad, F1 1.2× as long as broad, F2 2.36× as long as broad, F3 2.36× as long as broad, F4 2.36× as long as broad, clava 3-segmented 5.6× as long as broad. F1 0.5× as long as length of F2. F2 0.91× as long as F3. F3 = F4. Clava 2.4× as long as F4. Whorls of F1 reaching 1/3 basal part of F2, whorls of F2 reaching base of F3, whorls of F3 reaching base of F4. Whorls of F4 reaching base of C2 and whorls of C1 reaching apex of C3.

Mesosoma (Figure 5A,B). Mesoscutum 1.2–1.3× as broad as long, with distinct median line and 5 adnotaular setae in one row from both lateral sides. Scutellum 1.3× as broad as long. Propodeum 7.5× as broad as long, superfacial reticulate, callus with four setae in first row and 2 setae in the second row (Figure 5B).

Metasoma. Petiole small and transverse. Gaster (Figure 6B) 2.0× as long as broad; last tergite (Figure 6D) 5.7× as broad as long. Cercus with two setae (dorsal), the longest seta kinked, and 2.35× longer than next cercal seta (Figure 6D). The genitalia is slightly exerted (Figure 6B). The length of genitalia is 214.77 ± 6.63 μm (n = 8), ratio aedeagus to phallobase 1:2. Apodeme (Figure 7B) of aedeagus do not reach tip of phallobase (n = 8). Parameres on both sides of the aedeagus with two setae (Figure 7B), digiti with one developed spine. Ratio between apodeme of aedeagus and aedeagus 2.25:1 (Figure 7D).

Color of the male is similar to female except gaster. Gaster dark brown with yellow spot on Gt1-Gt3 (Figure 2B,D and Figure 6B).

Material. Holotype: ♀, China: Hechi City, reared from *O. bipolaris* on *E. urophylla* × *E. grandis* (DH32-28), 7-VIII-2023.

Paratypes. 196 ♀ and 295 ♂ same data as holotype (deposited in GXU).

Distribution. China (Guangxi).

Etymology. The species is named after its host, bipolaris. The Chinese name for it is 桉树叶瘿长尾啮小蜂 (eucalyptus torymid wasps).

Comments. The identification key of the European *Aprostocetus* is published by Graham [23]. *Aprostocetus bipolaris* sp. nov. is similar to *A. micantulus* (Thomson) and runs to couplets 174 (-). To include the new species in the key subgenus *Aprostocetus* (includes 218 species), the following changes can be made:

Females

174. Antenna with funicle slightly more slender … first funicular segment 1.5–2.0× as long as broad……………………………………….…………….*A. micantulus* (Thomson)
174a. Antenna. F1 = F2. F3 0.81× as long as F2.Clava 2.5× as long as F3 and 2.2× as long as broad. Gaster 1.2× as long as broad……….……….……….*A. micantulus* (Thomson)
- (a). Antenna. F1 = F2. F3 0.88× as long as F2. Clava 2.1× as long as F3 and 2.0× as long as broad. Gaster 1.6× as long as broad……………………*Aprostocetus bipolaris* sp. nov.

Males (Includes 111 species)

110. Spur of mid tibia fully as long as, or even very slightly longer than basitarsus. Antenna. Species associated with *Pinus* spp………………………………*A. micantulus*
-. Spur of mid tibia 0.87–0.90× length of basitarsus in *A. aethiops*, virtually as long as basitarsus in *A. lycidas* mostly on deciduous trees or shrubs………….……..*A. aethiops*
110a. Antenna with ventral plaque 0.29 of length of scape. F1 0.54× as long as F2. F2 0.91× as long as F3. F2 = F3. Whorls of F1 reaching level with tip of F3. Ratio between apodemae of aedeagus and aedeagus 2.2: 1. Digitus with 1 spine. Parasitoid of *Dasyneura abietiperda* Henschel [46] (Domenichini 1966: 39)….*A. micantulus* (Thomson)
-. (a) Antenna with ventral plaque 0.33 of length of scape. F1 0.5× as long as F2. F2 0.91× as long as F3. F3 = F4. Whorls of F1 reaching 1/3 basal part of F2. Ratio between apodemae of aedeagus and aedeagus 2.25: 1. Digitus with 1 spine. Parasitoid of *Ophelimus bipolaris* (Hymenoptera, Eulophidae)…………..….*Aprostocetus bipolaris* sp. nov.

Key to species of subgenus *Aprostocetus* (*Aprostocetus*) (Kostjukov: 347 in book: “Key to the insects of Russian Far East (ed. P.A. Lehr)”. 1995: 1–600. (Includes 117 species in one key for both sexes) [47]. Using this key our species runs to couplet:

8. Gaster 4.3–5.3× as long as broad………………….……….…*A.* (*A.*) *aquaticus* Erdoes
-. Gaster 1.7–4.0× as long as broad…………………….………….……….…….……….9
9. The longest cercal seta 1.5× longer than next cercal seta……*A.* (*A.*) *escherichi* Szelyni
-. The longest cercal seta 2.0× longer than next cercal seta; gaster 1.5–2.7× as long as broad; scutellum 1.15–1.3× as long as broad….……..….………*A.* (*A.*) *zosimus* Walker
9a. Antenna female F1 3.0× as long as broad, F2 2.0× as long as broad. Antenna male with ventral plaque 0.6 of length of scape. F1 0.5× as long as F2. F2 = F3 = F4. Whorls of F1 reaching 2/3 basal part of F3. Digitus with 3 spines. Parasitoid of *Dasyneura leguminicola* Lint (Cecidomyidae)………………………….….…..*A.* (*A.*) *zosimus* Walker
(a). Antenna female F1 1.9× as long as broad, F2 1.9× as long as broad. Antenna male with ventral plaque 0.33 of length of scape. F1 0.5× as long as F2. F2 0.91× as long as F3. F3 = F4. Whorls of F1 reaching 1/3 basal part of F2. Digitus with 1 spine. Parasitoid of *Ophelimus bipolaris* (Hymenoptera, Eulophidae)…...….*Aprostocetus bipolaris* sp. nov.

### 3.2. Phylogenetic Analyses

**Figure 8:**
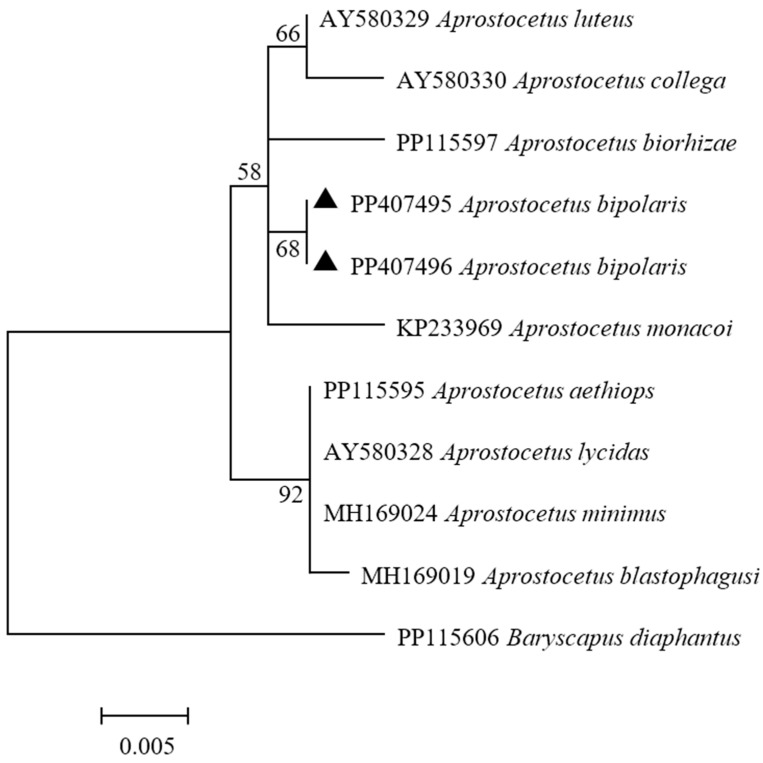
Maximum likelihood tree based on *28S*. The number at each branch indicates the percentage supported by bootstrap.

The *28S* rRNA gene was successfully amplified using PCR. The amplified products from one male and one female specimen were about 600 bp. The NCBI Nucleotide Blast tool was used to search *28S* rRNA sequences from various species. Among these sequences, *A. aethiops* (PP115595) and *A. biorrhizae* (PP115597), exhibited the highest sequence similarity to the target sequences (Table 1), both reaching 99.28%. Based on phylogenetic analysis, the parasitoid of *A. bipolaris* sp. nov. had an unambiguously independent position within the genus *Aprostocetus* clade (Figure 8).

**Figure 9:**
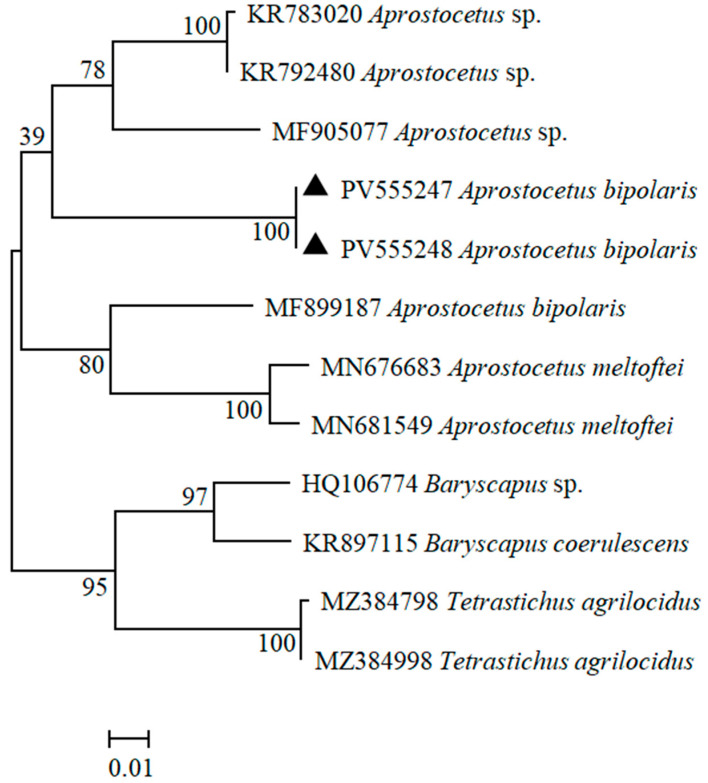
Maximum likelihood tree based on *COI*. The number at each branch indicates the percentage supported by bootstrap.

For *COI* gene, the sequences of the two specimens were identical, each with a length of 651 bp (Table 1). An initial similarity analysis of the sequences showed that the obtained sequences exhibited the highest similarity of 90.54% with *Aprostocetus* sp. (KR783020) from Canada in the GenBank database. Phylogenetic analysis showed that *A. bipolaris* sp. nov. clustered together with other species within genus *Aprostocetus* in the same major clade, indicating a close evolutionary relationship (Figure 9). However, *A. bipolaris* sp. nov. further formed an independent and distinctly differentiated subclade within this major clade. These findings suggest *A. bipolaris* sp. nov. belongs to the genus *Aprostocetus* and is genetically different from other congeneric species.

### 3.3. Biology

*Aprostocetus bipolaris* sp. nov. was reared from galls of *O. bipolaris* on *E. urophylla* × *E. grandis* in Guangxi, China. Adults collected during August 2023 emergence (n = 491 specimens) showed a male-biased sex ratio (♀:♂ = 1:1.51; 196♀/295♂) and 18.52% parasitoidism rate. While emergence was documented in August, seasonal occurrence requires further study. This parasitoidism rate suggests potential biocontrol utility against *O. bipolaris*.

## 4. Discussion

*Aprostocetus* is a biologically and morphologically highly diverse genus, with a worldwide distribution. However, species identification keys for this genus are mostly available for Europe [23] and India [48,49]. Moreover, the lack of DNA sequences for certain species further complicates taxonomic identification. Consequently, synonymies may be identified in future revisions. However, based on the current dataset, morphological and molecular examination of the unknown parasitoid from genus *Aprostocetus* shows that it is a previously unrecorded species. In this study, we recorded it as a new species, *A. bipolaris* sp. nov., in combination with its host.

*Aprostocetus bipolaris* sp. nov. has a single row of adnotaular setae, malar sulcus present and complete, submarginal vein with three setae, one of the cereal setae distinctly longer than the remaining setae and kinked. These characteristics are consistent with the identifying features of *Aprostocetus* by LaSalle [24]. For *A. bipolaris* sp. nov., it is most similar to the Palearctic *A. micantulus*. However, females and males of the two species can be distinguished from each other based on running the key compiled by Graham [23]. Moreover, the hosts of the two species are different. Hosts of *A. micantulus* associated with *Pinus* spp., while *A. bipolaris* sp. nov. is a parasitoid of *O. bipolaris*. Kostjukov provided a key to subgenus *Aprostocetus* (*Aprostocetus*), including 117 species [47]. According to the key, *A. bipolaris* sp. nov. can be separated from most *Aprostocetus* species. Therefore, we ascertained that the parasitic wasp emerged from galls of *O. bipolaris* is a different species from the other *Aprostocetus* species.

Molecular identification is a valuable complement to morphological taxonomy. Our investigation inferred the relations of new species and other *Aprostocetus* species from *28S* and *COI* genes. For the *28S* gene sequence, *A. bipolaris* sp. nov. has a high similarity to *A. biorrhizae* and *A. aethiops*. However, there are morphological differences between *A. bipolaris* sp. nov. and *A. aethiops*. For *A. bipolaris* sp. nov., the mid tibial spur is equal to or slightly longer than basitarsus, whereas in *A. aethiops*, the mid tibial spur is distinctly shorter (ratio: 0.87–0.90×). Additionally, the biological traits of *A. bipolaris* sp. nov. and *A. aethiops* are different. The hosts of *A. aethiops* are mainly associated with deciduous trees or shrubs, which also differentiates it from *A. bipolaris* sp. nov. [23]. *Aprostocetus biorrhizae* has the following characteristics [50]: (1) Abdomen with 3–6 cercal setae of similar length; (2) Middle lobe of the shield with a reticulated sculpture whose reticulum is 2–3 times longer than wide; (3) Dark metallic body, with more or less extended areas of intense yellow coloring. Moreover, *A. biorrhizae* is a parasitoid of *Biorrhiza pallida* and *Dryocosmus kuriphilus*. These two species infest *Castanea sativa*, *Quercus faginea*, and *Quercus petraea* as their host plant.

Molecular phylogenetic analyses based on *28S* and *COI* sequences revealed that *A. bipolaris* sp. nov. forms a distinct monophyletic clade, clearly separated from other *Aprostocetus* species. This result confirms its taxonomic placement within *Aprostocetus* while demonstrating significant genetic divergence from congeners. The congruence between molecular and morphological evidence supports the recognition of *A. bipolaris* sp. nov. as a new species within *Aprostocetus*.

According to the collected sample data, the *A. bipolaris* sp. nov. population exhibits a male-biased sex ratio (ca. 60% males). This finding differs from observations of other *Aprostocetus* species [36,51]. This might be related to the dispersal strategy of the new species [52]. *Ophelimus bipolaris,* as a new invasive species of the *Eucalyptus* gall wasp in China, currently lacks effective control methods. This parasitoid, an important biological control agent, is considered to have long-term ecological and economic benefits in terms of controlling exotic pests [15]. However, the number of parasitoid species of *O. bipolaris* and their parasitic capacities in the field in China are still unknown. Our findings indicate that *A. bipolaris* sp. nov. can be applied for the biological control of *O. bipolaris* and potentially other *Eucalyptus* gall wasps species, although further investigation is required to confirm its efficacy.

## Figures and Tables

**Table 1:** GenBank accession numbers.

Sex	Accession Number	
	*28S*	*COI*
Female	PP407495	PV555247
Male	PP407496	PV555248

## Data Availability

The original contributions presented in the study are included in the article; further inquiries can be directed to the corresponding author.
